# Case Report: Focal cryoablation vs. radiofrequency ablation in a pediatric patient with para-Hisian accessory pathway with effect from the non-coronary aortic cusp

**DOI:** 10.3389/fped.2024.1405104

**Published:** 2024-08-20

**Authors:** Zhandos Maksut, Raushan Zhanbolatkyzy, Abay Bakytzhanuly, Almira Bajgalkanova, Yakup Ergül, Omirbek Nuralinov

**Affiliations:** Department of Interventional Arrhythmology, University Medical Center, Astana, Kazakhstan

**Keywords:** case report, non-coronary cusp, Wolff–Parkinson–White syndrome, radiofrequency ablation, anteroseptal accessory pathway, para-Hisian accessory pathway, pediatric patient

## Abstract

A 12-lead electrocardiogram of a pediatric patient with Wolff–Parkinson–White syndrome was consistent with the anteroseptal accessory pathway. The patient had three ablation procedures because of the recurrences of the arrhythmia. In our case, successful cryoablation was performed in the non-coronary cusp of the aortic root.

## Introduction

Wolff–Parkinson–White (WPW) syndrome is the predominant type of the tachycardia observed in pediatric patients, requiring electrophysiology study and ablation (1). Various algorithms have been developed, which are based on QRS morphology in electrocardiogram (ECG), to increase efficiency and predict accessory pathway (AP) locations in patients with WPW syndrome (2). Ablation of septal accessory pathways has a potential risk of injury owing to their proximity to the compact atrioventricular (AV) node, His bundle. The ablation of anteroseptal accessory pathways may require an alternative approach to enhance catheter contact and ablation success. In rare cases, non-coronary cusp is such an approach (3). Reversible effects of the cryoablation at relatively lower temperatures are increasingly used to create safe and effective ablation (4). The aim of our case report was to reveal the possible site of ablation of the para-Hisian accessory pathway.

## Case report

**Figure 1:**
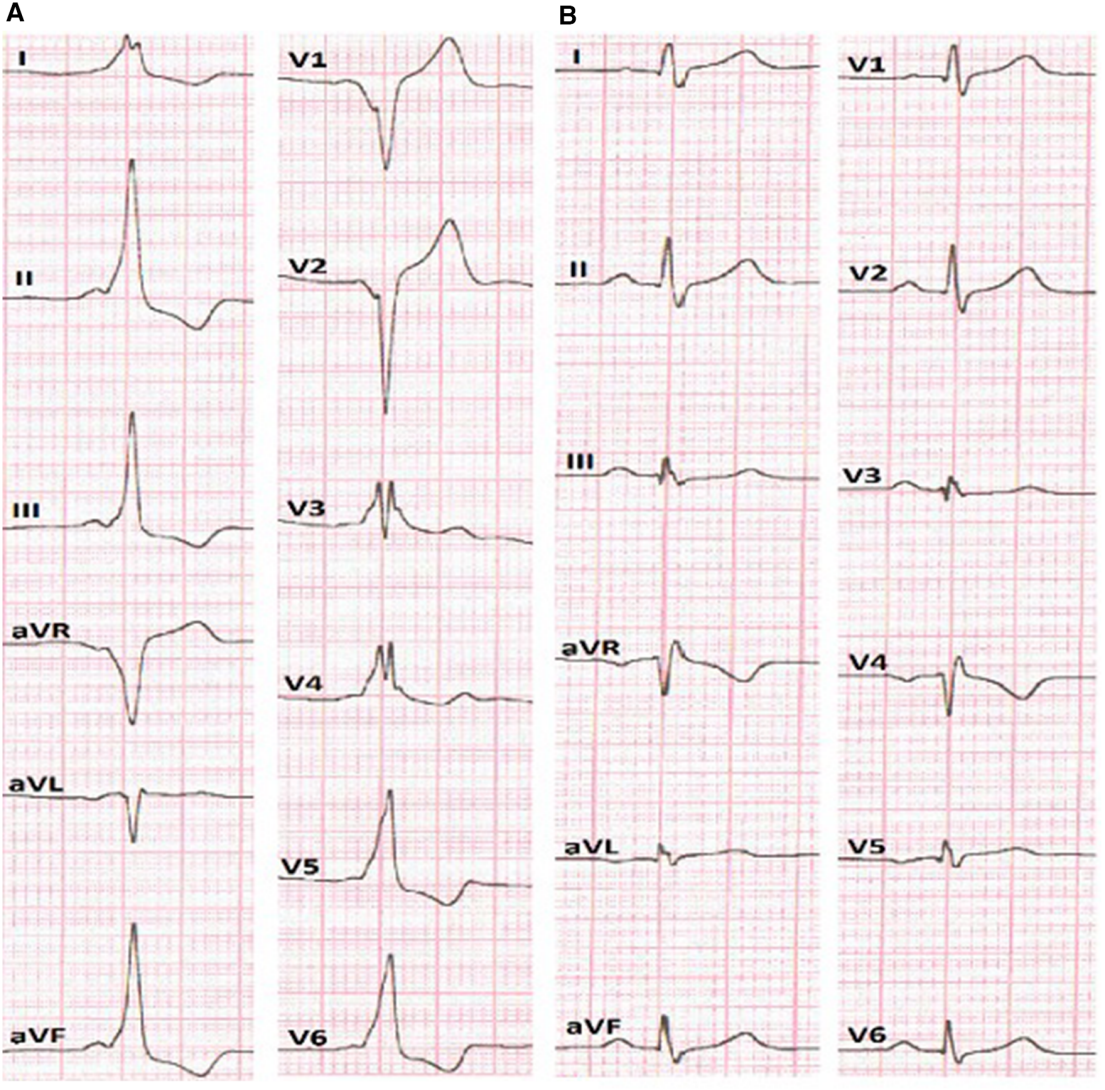
**(A)** ECG before the RFA presumes anteroseptal localization of AP. **(B)** ECG after the RFA without signs of pre-excitation of the ventricles.

A 15-year-old male patient, with a height of 176 cm and weight of 66 kg, was referred to our clinic because of episodes of palpitations. On surface electrocardiogram registered paroxysm of supraventricular tachycardia (SVT) with a cycle length of 320 milliseconds (ms). Physical examination, laboratory test results, and echocardiographic parameters were normal. The surface ECG indicated the presence of an anteroseptal accessory pathway (Figure 1A) using the EASY-WPW algorithm (5). All anti-arrhythmic drugs were stopped for a minimum of five half-lives before the procedure. After informed consent was obtained, an electrophysiological (EP) study was performed under local anesthesia. The navigation system EnSiteX (Abbott, USA) was used for local activation time (LAT) mapping.

After obtaining femoral vascular access, a decapolar diagnostic catheter was placed into the coronary sinus, while other mapping catheters were placed at the high right atrium (HRA) and right ventricular apex position. Basic electrophysiological data revealed an AH interval (conduction time from the low-right atrium at the interatrial septum through the AV node to the His bundle) of 59 ms and HV interval (conduction time from the proximal His bundle to the ventricular Myocardium) of 36 ms. The AP effective refractory period was 290 ms. The presence of a para-Hisian AP was demonstrated during antegrade LAT mapping in sinus rhythm. The earliest ventricular signals were detected near the bundle of His. Radiofrequency ablation (RFA) was performed in the right anteroseptal region, leading to a reduction in ventricular pre-excitation from coronary sinus (CS) 9–10 to CS 7–8. Subsequently, through a transseptal puncture, an ablation catheter was inserted into the left atrium. Mapping of the earliest ventricular signal was performed around the mitral annulus. LAT mapping revealed the earliest point in the left anteroseptal region with a 30 ms lead before the onset of the delta wave. RF energy titration (from 25 to 35 W, 43 °C) led to the loss of pre-excitation after the fourth attempt without any complications. After ablation, there was no recurrence of pathway conduction, and atrioventricular reentrant tachycardia (AVRT) was no longer inducible (Figure 1B).

However, recurrence of the clinical symptoms, without pre-excitation signs, occurred 1 month after RFA was performed.

The patient was rehospitalized and examined, and laboratory and instrumental examination data were normal. For the EP study, we used the Ensite Precision system (Abbott, USA). Ablation and decapolar catheters were inserted via the left (introducer 6f) and right (introducer 7f) femoral veins. No pre-excitation signs were observed on the decapolar catheter electrogram. Ante- and retrograde pacing did not induce any jumps, echoes, or tachycardias. Isoprenaline infusion followed by antegrade programmed pacing induced SVT with a tachycardia cycle length (TCL) of 365 ms (post-pacing interval (PPI) − TCL = 470 − 375 = 105 ms; stimulus-atrial interval (SA) − ventriculoatrial interval (VA) = 180 − 127 = 53 ms). The administration of 15 mg adenosine revealed only retrograde conduction of the accessory pathway and revealed VA fusion on CS 9–10 electrogram. LAT mapping during AVRT identified the accessory pathway in the right anteroseptal region. The transjugular approach was preferred for ablation considering the prior transfemoral RFA. An irrigated catheter [TactiCath (Abbott, USA)] was inserted into the right atrium. Ablation (30–35 W, 40 °C, impedance 125 Ω) was performed under the control of AV conduction. SVT was eliminated after four lesions.

However, retrograde pacing revealed an eccentric atrial activation pattern with CS 7–8 leading. LAT mapping after the transseptal approach showed the earliest point in the left anteroseptal region. After RFA was performed, the form of CS changed to concentric.

Programmed pacing after isoprenaline infusion induced SVT with a TCL of 300 ms (PPI − TCL = 500 − 300 = 200 ms; SA − VA = 225 − 90 = 135 ms). Subsequent tests confirmed the last SVT as typical atrioventricular nodal reentrant tachycardia (AVNRT), leading to the ablation of the “slow pathway.” No recurrences of pathway conduction or inducible jumps, echoes, or tachycardia were observed after RFA.

After 3 months, the patient was readmitted due to the recurrences of the palpitations after the two previous RFA procedures.

**Figure 2:**
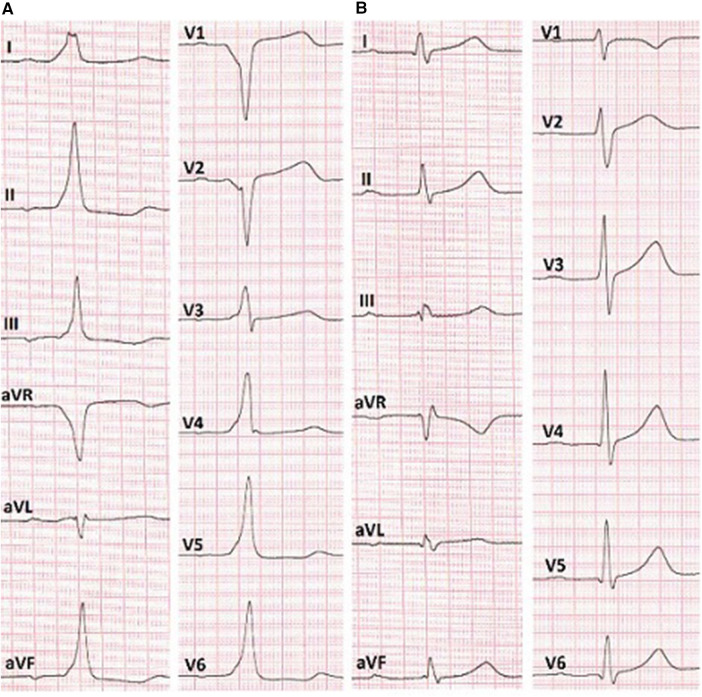
**(A)** ECG before the RFA shows anteroseptal AP localization (EASY-WPW algorithm). **(B)** ECG after the cryoablation without signs of pre-excitation of the ventricles.

In the surface electrocardiogram (Figure 2A), pre-excitation was observed with a P-R interval lasting 150 ms. The delta wave had a positive pattern in leads I and II, while in lead V1, the S wave was deeper, indicating the presence of a right-sided accessory pathway. However, the resulting axis for the delta wave showed greater positivity in lead II compared to lead aVL. This pattern suggested the presence of a right anteroseptal accessory pathway (EASY-WPW algorithm).

The navigation system EnsiteX was used for LAT mapping. Basic electrophysiological data revealed an AH interval of 60 ms and an HV interval of 34 ms. The AP effective refractory period was 310 ms. During the study, antegrade programmed pacing led to the induction of paroxysmal orthodromic atrioventricular tachycardia. Subsequently, retrograde pacing mapping identified the AP in the para-Hisian region. The earliest ventricular electrogram was 28 ms before the onset of the delta wave.

Based on the anatomical relationship between the para-Hisian region and non-coronary cusp, mapping in the aortic cusp was performed via the right femoral artery.

To diagnose the condition, a diagnostic/ablation catheter was inserted into the aorta. Subsequently, we positioned catheters sequentially in the right coronary cusp, left coronary cusp, and the non-coronary cusp of the aortic valve. In the non-coronary cusp, we observed a site of atrial activation occurring 13 ms earlier (−41 ms) than the electrograms recorded in the right atrium.

**Figure 3:**
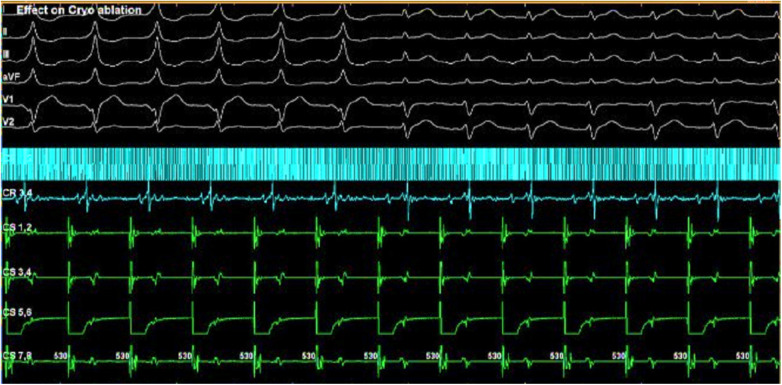
Effect during the focal cryoablation from the non-coronary aortic cusp.

**Figure 4:**
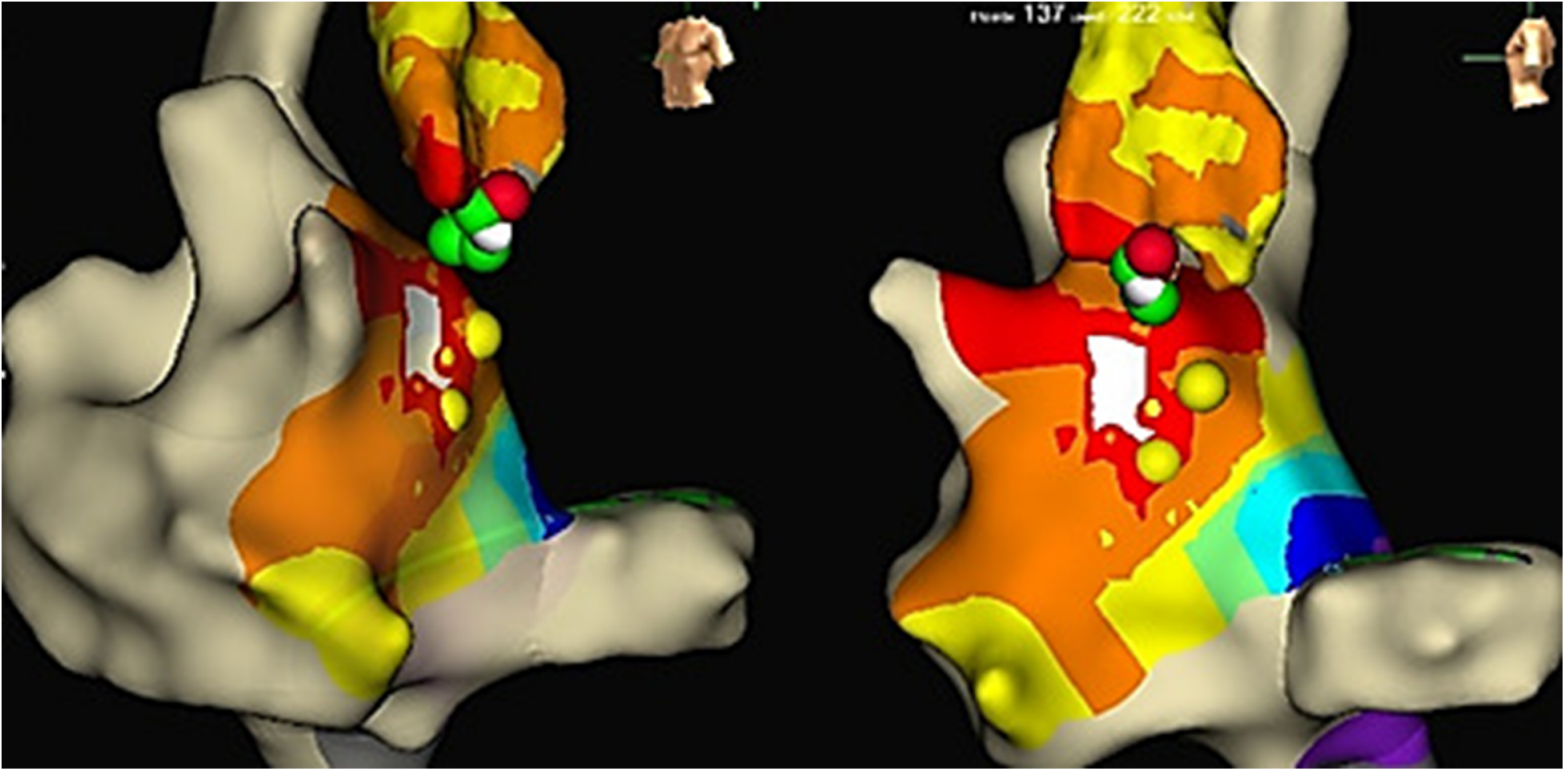
LAT mapping in the aortic cusps. The earliest activation was observed in the non-coronary cusp. Yellow colored points, His bundle localization; red and green colored points, lesions from the non-coronary cusp.

Reversible effects of the cryoablation at lower temperatures (6), previously performed RFA attempts in the para-Hisian region, and risk of atrioventricular block made this method preferable to RFA. We used a Medtronic Freezor catheter (Minneapolis, USA) with a 7 Fr diameter and a 6 mm tip, which was inserted into the aortic root for focal cryoablation. Under the control of the X-ray imaging and navigation system, we conducted cryomapping in the area with the earliest ventricular pre-excitation. In the non-coronary cusp, focal cryoablation was performed for a duration of 480 s using parameters set at −30 °C to ensure that conduction through the AV node remained unaffected. The accessory pathway was ablated and abolished during the second application (Figures 2B, 3). A total of five applications were applied, achieving a temperature drop to −74 °C (Figure 4).

After the ablation procedure, there were no recurrences of pathway conduction or inducible orthodromic reciprocating tachycardia. In addition, adenosine testing revealed the absence of conduction through the accessory pathway.

## Discussion

Radiofrequency ablation is the treatment of choice for symptomatic patients with accessory pathways. Anteroseptal accessory pathways may be located at the anterior membranous septum or may have an epicardial localization. The aortic non-coronary cusp is an alternative access point, especially for the accessory pathways that cannot be ablated from anteroseptal region. However, the high risk of damage to the His bundle and the AV node during RFA of accessory pathways makes cryoablation a potential option to reduce the atrioventricular block risks. In our case, we found the earliest ventricular pre-excitation while mapping the non-coronary cusp with epicardial localization. After the ablation of this site, pre-excitation disappeared.

In conclusion, with para-Hisian accessory pathways, if ablation is not possible on the right atrial side, the non-coronary aortic cusp may provide an alternative ablation approach.

## Data Availability

The original contributions presented in the study are included in the article/Supplementary Material, further inquiries can be directed to the corresponding authors.
